# 4-Hexylresorcinol induced angiogenesis potential in human endothelial cells

**DOI:** 10.1186/s40902-020-00267-2

**Published:** 2020-06-29

**Authors:** Min-Keun Kim, Seong-Gon Kim, Suk Keun Lee

**Affiliations:** 1 Department of Oral and Maxillofacial Surgery, College of Dentistry, Gangneung-Wonju National University, and Institute of Oral Science, 123 Chibyun-dong, Gangneung, 210-702 Republic of Korea; 2 Department of Oral Pathology, College of Dentistry, Gangneung-Wonju National University, and Institute of Oral Science, 123 Chibyun-dong, Gangneung, 210-702 Republic of Korea

**Keywords:** 4HR, HUVEC, IP-HPLC, TGF-β1, Angiogenesis

## Abstract

**Background:**

4-Hexylresorcinol (4HR) is able to increase angiogenesis. However, its molecular mechanism in the human endothelial cells has not been clarified.

**Methods:**

As endothelial cells are important in angiogenesis, we treated the human umbilical vein endothelial cells (HUVECs) with 4HR and investigated protein expressional changes by immunoprecipitation high-performance liquid chromatography (IP-HPLC) using 96 antisera.

**Results:**

Here, we found that 4HR upregulated transforming growth factor-β (TGF-β)/SMAD/vascular endothelial growth factor (VEGF) signaling, RAF-B/ERK and p38 signaling, and M2 macrophage polarization pathways. 4HR also increased expression of caspases and subsequent cellular apoptosis. Mechanistically, 4HR increased TGF-β1 production and subsequent activation of SMADs/VEGFs, RAF-B/ERK and p38 signaling, and M2 macrophage polarization.

**Conclusion:**

Collectively, 4HR activates TGF-β/SMAD/VEGF signaling in endothelial cells and induced vascular regeneration and remodeling for wound healing.

## Background

4-Hexylresorcinol (4HR) is a substituted phenol that is synthesized from resorcinol and caproic acid [1]. It is used as an antimicrobial in tooth pastes and skin lotions [2] and as a preservative for fresh fruits and vegetables [3]. *It has* bactericidal [4], anthelmintic [5], and potential antineoplastic activities [6], and thus, it is also used as an antiseptic in mouthwashes and skin wound cleansers [7]. 4HR may also inhibit oxidative DNA damage by enhancing the activities of antioxidant enzymes, including glutathione peroxidase and glutathione reductase, which facilitate the scavenging reactive oxygen species by glutathione [8], and thus, it is also used to prevent the enzymatic browning of shrimps and different fruits [9].

A recent study demonstrated that 4HR increases the expression level of vascular endothelial growth factor (VEGF) in RAW264.7 cells and angiogenesis in the animal model [10]. 4HR increases M2 markers, and broad-spectrum matrix metalloproteinase (MMP) inhibitor (PD166793) can reduce 4HR-induced VEGF expression. However, MMPs are also highly expressed in the inflammatory phase, and the expression of MMPs is mostly regulated by hypoxic stress [11]. Interestingly, the action of PD166793 is mediated by chelating zinc ion [12]. Accordingly, zinc-dependent protein like transforming growth factor-β1 (TGF-β1) may be regulated by 4HR and induce VEGF and angiogenesis.

Immunoprecipitation high-performance liquid chromatography (IP-HPLC) had been used previously by several authors to detect organic compounds quantitatively, including peptides, but the technique used was complicated and of limited applicability [13, 14]. Recently, a new IP-HPLC protocol was developed to determine protein expression levels in different biological fluids, such as blood serum, urine, saliva [15], inflammatory exudates [16–18], and different protein extracts from cells [19–21], liver [22], and cancer tissues [21]. Recent IP-HPLC results demonstrate that 4HR administration increases the expression of TGF-β1 in the osteoblast-like cells [23]. IP-HPLC is comparable to enzyme-linked immunosorbent assay (ELISA), but the former uses protein A/G agarose beads in buffer solution and ultraviolet spectroscopy to determine protein concentrations, whereas the latter uses fluorescence-conjugated antibodies fixed in plastic wells and fluoroscopy. Furthermore, multiple trials have shown that IP-HPLC can be used to rapidly determine multiple protein levels accurately (± 5% standard deviation) and reproducibly.

In this study, differentially expressed proteins by 4HR were screened by IP-HPLC in a human endothelial cell line (human umbilical vein endothelial cells [HUVECs]) using our antibody library. IP-HPLC results demonstrated that TGF-β1 played a key role in 4HR-induced activation of angiogenesis-associated signal pathway in HUVEC cells. To confirm this hypothesis, additional western blotting was done with TGF-β1 and its signal blocker.

## Methods

### HUVEC culture in the presence of 4HR

HUVECs (Lonza, Walkersville, MD, USA) were purchased and cultured in an endothelial basal medium supplemented with 1 μg/mL hydrocortisone, 12 μg/mL bovine brain extract, 50 μg/mL gentamicin, 50 ng/mL amphotericin-B, 10 ng/mL epidermal growth factor (EGF), VEGF, FGF-2, heparin, ascorbic acid, and 10% fetal calf serum (EGM^TM^-2, Clonetics®, Lonza, Walkersville, MD, USA) in 5% CO_2_ at 37.5 °C. Cells were tested for mycoplasma on a regular basis to ensure that only mycoplasma-free cells were assayed.

About 70% confluent HUVECs grown on Petri dish surfaces were treated with 10 μg/mL 4HR (with a single dose given safely given in dog; 100–300 mg/kg, WHO food additives Series 35, 835) for 8, 16, or 24 h; control cells were treated with 1 mL of normal saline. Cultured cells were harvested with protein lysis buffer (PRO-PREP^TM^, iNtRON Biotechnology INC, Korea) and immediately preserved at − 70 °C until required.

### Immunoprecipitation high-performance liquid chromatography (IP-HPLC)

Protein extracts (100 μg) were subjected to immunoprecipitation using a protein A/G agarose column (Amicogen, Korea). Protein A/G agarose columns were separately pre-incubated with 1 μg of 96 different antisera for growth factor-related proteins (*n* = 10), RAS signaling proteins (*n* = 22), NFkB signaling proteins (*n* = 12 [2]), apoptosis-related proteins (*n* = 20), inflammatory proteins (*n* = 20), angiogenesis-related proteins (*n* = 14 [3]), and control housekeeping proteins (*n* = 3) (numbers in brackets indicate the number of overlapping antibodies; Table 1).

**Table 1:** Antibodies used in the study

Protein	No.	Antibodies
Growth factor-related protein	10	FGF-1*, FGF-2*, CTGF, TGF-β1^#^, TGF-β2*, TGF-β3*, SMAD4*, SMAD2/3, p-SMAD4, PDGF-A*
RAS signaling proteins	22	NRAS^$^, KRAS^$^, HRAS, PI3K, pAKT1/2/3, RAF-B*, JNK-1*, p-JNK-1, ERK-1*, p-ERK-1^$^, Rab 1*, STAT3, p38*, p-p38*
NFkB signaling proteins	12 (2)	NFkB*, IKK*, GADD45*, GADD153*, mTOR^@^, NRF-2*, PGC-1α, SRC-1*, MDR, AMPK (p38*, p-p38*)
Inflammatory proteins	20	IL-10*, lysozyme*, granzyme, lactoferrin, M-CSF, Pdcd-1/1, HCAM, ICAM-1, COX2*, versican, TNFα^@^, IL-6*, LTA4H^&^, CXCR4, cathepsin C, cathepsin G*, MCP-1, CD68, CD99, TLR3
Apoptosis-related proteins	20	p53*, BAD*, BAK*, BAX, BCL2, AIF*, APAF-1, caspase 9*, c-caspase 9*, PARP-1*, c-PARP-1*, FASL*, FAS*, FADD*, FLIP*, BID, c-caspase 8*, c-caspase-10, caspase 3*, c-caspase 3*
Angiogenesis-related proteins	14 (3)	HIF-1α^&^, angiogenin^$^, VEGF-A*, VEGF-C*, vWF^$^, CMG2^$^, FLT-4^$^, LYVE-1*, MMP-2, MMP-10, PECAM-1 (FGF-2, PDGF-A, ICAM-1)
Control housekeeping proteins	3	α-Tubulin*, β-actin*, GAPDH*
Total	101 (5)	

The number of antibodies that overlapped is indicated in parentheses

*Abbreviations*: *AIF* apoptosis inducing factor, *AMPK* AMP-activated protein kinase, *pAKT* v-akt murine thymoma viral oncogene homolog, p-Akt1/2/3 phosphorylated (p-Akt, Thr 308), *BAD* BCL2-associated death promoter, *BAK* BCL2 antagonist/killer, *BAX* BCL2-associated X, *CMG2* capillary morphogenesis protein 2, *COX-2* cyclooxygenase-2, *CTGF* connective tissue growth factor, *CXCR4* C-X-C chemokine receptor type 4, *FADD* FAS-associated via death domain, *FAS* CD95/Apo1, *FASL* FAS ligand, *FGF-1* fibroblast growth factor-1, *FLIP* FLICE-like inhibitory protein, *FLT-4* Fms-related tyrosine kinase 4, *GADD45* growth arrest and DNA damage-inducible 45, *GAPDH* glyceraldehyde 3-phosphate dehydrogenase, *HCAM* (CD44) homing cell adhesion molecule, *HDAC-10* histone deacetylase 10, *HIF-1α* hypoxia-inducible factor-1α, *HRAS* GTPase HRas, *HSP-70* heat shock protein-70, *ICAM* (*CD54*) intercellular adhesion molecule 1, *IKK* ikappaB kinase, *IL-1* interleukin-1, *JNK-1* Jun N-terminal protein kinase, *KRAS* V-Ki-ras2 Kirsten rat sarcoma viral oncogene homolog, *LTA4H* leukotriene A4 hydrolase, *LYVE-1* lymphatic vessel endothelial hyaluronan receptor 1, *MCP-1* monocyte chemotactic protein 1, *M-CSF* macrophage colony-stimulating factor, *MDR* multiple drug resistance, *MMP-2* matrix metalloprotease-2, *mTOR* mammalian target of rapamycin, *NCAM* (*CD56*) neural cell adhesion molecule 1, *NF-1* neurofibromin 1, *NFkB* nuclear factor kappa-light-chain-enhancer of activated B cells, *NRAS* neuroblastoma RAS viral oncogene homolog, *NRF2* nuclear factor (erythroid-derived)-like 2, *PARP-1* poly-ADP ribose polymerase 1, *c-PARP-1* cleaved PARP-1, *Pdcd-1/1* (*CD279*) programmed cell death protein 1, *PDGF-A* platelet-derived growth factor-A, *PECAM-1* (*CD31*) platelet endothelial cell adhesion molecule-1, *PGC-1α* peroxisome proliferator-activated receptor gamma coactivator 1-α, *PI3K* phosphatidylinositol-3-kinase, *PTEN* phosphatase and tensin homolog, *Rab 1* Rab GTPases, *RAF-B* v-Raf murine sarcoma viral oncogene homolog B, *SMAD4* mothers against decapentaplegic, drosophila homolog 4, *SRC1* steroid receptor coactivator-1, *STAT3* signal transducer and activator of transcription-3, *TGF-β1* transforming growth factor-β1, *TNFα* tumor necrosis factor-α, *VEGF-A* vascular endothelial growth factor A, *vWF* von Willebrand factor

*Santa Cruz Biotechnology, CA, USA

^#^DAKO, Denmark

^$^Neomarkers, CA, USA

^@^ZYMED, CA, USA

^&^Abcam, Cambridge, UK

Briefly, protein samples were mixed with 5 mL of binding buffer (150 mM NaCl, 10 mM Tris pH 7.4, 1 mM EDTA, 1 mM EGTA, 0.2 mM sodium vanadate, 0.2 mM PMSF, and 0.5% NP-40) and incubated in protein A/G agarose (Amicogen, Korea) columns on a rotating stirrer for 1 h at 4 °C. After washing columns with PBS (phosphate-buffered saline solution), target proteins were eluted using 150 μL of IgG elution buffer (Pierce, USA). Immunoprecipitated proteins were analyzed using an HPLC unit (1100 series, Agilent, USA) equipped with a reverse phase column and a micro-analytical detector system (SG Highteco, Korea). Elution was performed using 0.15M NaCl/20% acetonitrile solution at 0.4 mL/min for 30 min, and proteins were detected using an ultraviolet spectrometer at 280 nm. Control and experimental samples were run sequentially to allow comparisons. For IP-HPLC, whole protein peak areas (mAU*s) were calculated after subtracting negative control antibody peak areas, and square roots of protein peak areas were calculated to normalize concentrations. Protein percentages in total proteins in experimental and control groups were plotted. Results were analyzed using the chi-squared test [19–21].

The housekeeping proteins *β*-actin, *α*-tubulin, and glyceraldehyde 3-phosphate dehydrogenase (GAPDH) were used as internal controls. Expressional changes of housekeeping proteins were adjusted to < ± 5% using a proportional basal line algorithm. Protein expressional changes of ≤ ± 5%, ± 5–10%, ± 10–20%, and ≥ ± 20% change were defined as minimal, slight, meaningful, or marked, respectively.

### Statistical analysis

Proportional data (%) of the experimental and control groups were plotted into line graphs and star plots, and analyses were repeated two to six times until standard deviations were ≤ ± 5%. Line graphs revealed the similarities of the expression pattern between the relevant proteins, and star plots revealed the differences in the expression levels of the whole objective proteins. Results were analyzed using the chi-squared test. The expressions of control housekeeping proteins, that is, *β*-actin, *α*-tubulin, and GAPDH, were nonresponsive (≤ 5%) to 12, 24, or 48 h of 4HR treatment.

## Results

### Effects of 4HR on the expressions of growth factor-related proteins in HUVECs

**Fig. 1:**
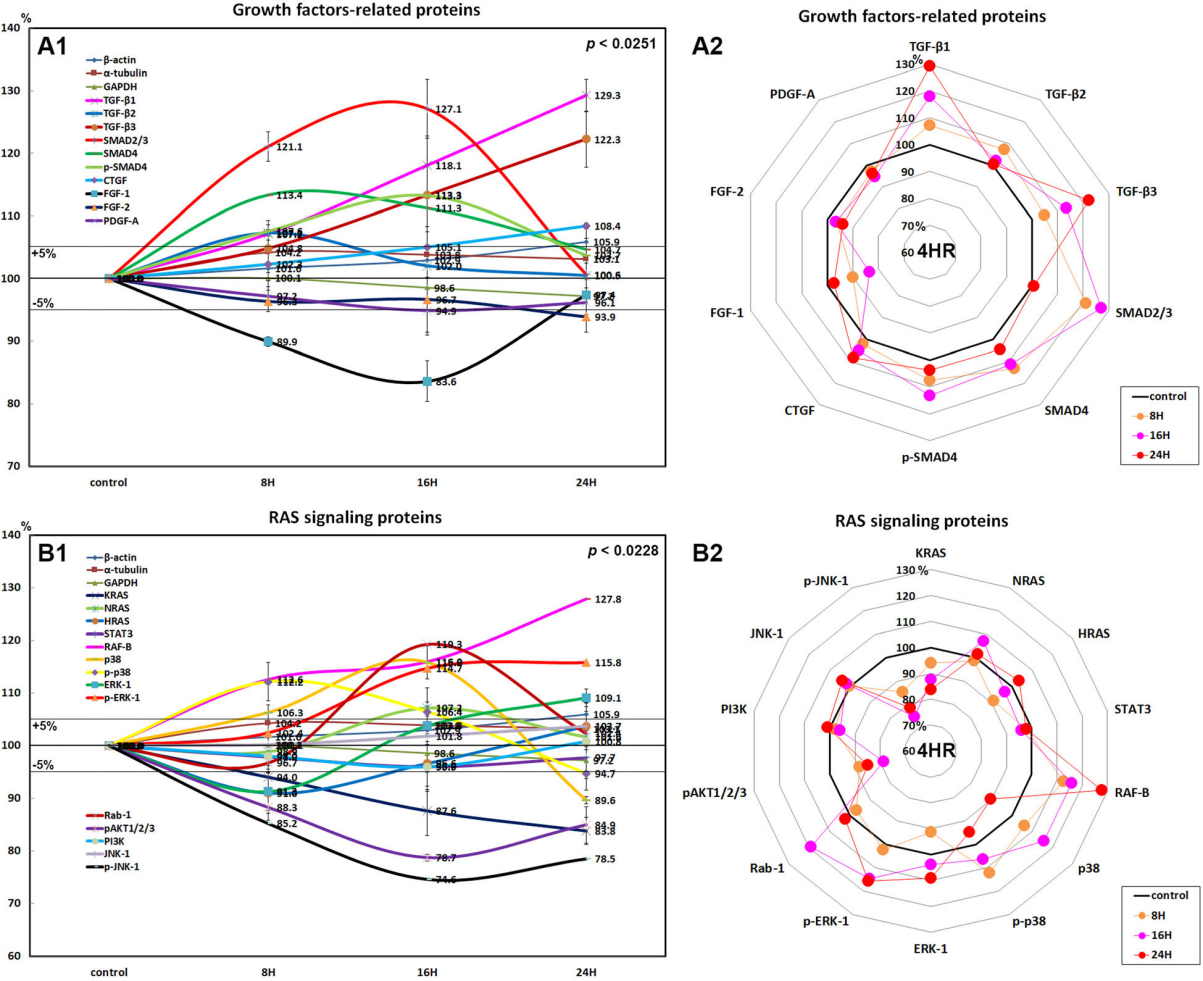
Expressions of growth factor-related proteins (**A1**, **A2**) and RAS signaling proteins (**B1**, **B2**) in 4HR-treated HUVECs as determined by IP-HPLC. The line graphs (**A1**, **B1**) show protein expression patterns on the same scale (%) versus culture time (8, 16, or 24 h), whereas the star plots (**A2**, **B2**) show the differential expression levels of proteins in a circular manner after 8, 16, or 24 h of treatment on appropriate scales (%)

HUVECs treated with 4HR showed marked increases in the expressions of TGF-β1 (29.3% at 24 h), TGF-β2 (7.3% at 8 h), TGF-β3 (22.3% at 24 h), SMAD2/3 (27.1% at 16 h), SMAD4 (13.4% at 8 h), and p-SMAD4 (13.3% at 16 h) and a slight increase in the expression of connective tissue growth factor (8.4% at 24 h) as compared with nontreated control, but a decrease in the expression of fibroblast growth factor-1 (FGF-1; 16.4% at 16 h), FGF-2 (6.1% at 24 h), and platelet-derived growth factor-A (PDGF-A; 5.1% at 16 h; Fig. 1A1, A2).

These results indicate 4HR increased the expressions of growth factors associated with TGF-β/SMAD pathways in HUVECs but slightly decreased the expressions of FGF-1, FGF-2, and PDGF-A. Therefore, we considered that 4HR provided dominant TGF-β-dependent angiogenesis in HUVECs despite downregulation of matrix angiogenetic factors (e.g., FGF-1, FGF-2, and PDGF-A).

### Effects of 4HR on the expressions of RAS signaling proteins in HUVECs

The expressions of RAS signaling proteins were variable in HUVECs treated with 4HR for 24 h. K-RAS expression gradually decreased by 16.2% at 24 h, H-RAS expression decreased by 9% at 8 h but increased by 3.7% at 24 h versus nontreated control, while N-RAS increased by 2% at 16 h and by 1.6% at 24 h. Downstream signal proteins SOS1/2 and STAT3 tended to be decreased by 11.3% and 5% at 16 h, respectively.

4HR upregulated RAF-B, a growth signal transduction protein kinase, by 27.8% at 2 h in HUVECs and subsequently upregulated mitogen-activated protein kinase 3, also known as extracellular signal-regulated kinase (ERK-1) and p-ERK-1 (Thr 202/Tyr 204) by 9.1% and 15.8% at 24 h, respectively. 4HR also upregulated p38 mitogen-activated protein kinase (p38, 15.8% at 16 h) and phosphorylated p38 (p-p38, 12.2% at 8 h). The critical mediator of growth factor-induced signals pAKT1/2/3 (Thr 308) was consistently downregulated by 21.3% at 24 h and by 15.1% at 8 h, and phosphorylated c-Jun N-terminal kinase-1 (p-JNK-1, 89; Thr 183/Tyr 185), which is responsible for stress stimuli, such as cytokinesultraviolet irradiation, heat shock, and osmotic shock, was also downregulated by 24.5% at 16 h and by 21.5% at 24 h, although the expression of non-phosphorylated JNK-1 was slightly increased by 3.7% at 24 h (Fig. 1)B1, B2). On the other hand, 4HR-treated HUVECs showed decreases in the expressions of protein kinase C (PKC; 18.6% at 8 h) and p-PKC (13.4% at 8 h) but minimal expression changes of phosphatidylinositol 3-kinase (PI3K), A-kinase anchoring proteins (AKAP), and phosphatase and tensin homolog (PTEN) by ± 5%. These results indicate that 4HR significantly upregulated the downstream effectors of RAS signaling, RAF-B/ERK-1 and p38 in HUVECs, while it attenuated other RAS signaling pathways (e.g., AKT and JNK signaling) and minimally affected the expressions of PI3K, AKAP, and PTEN.

### Effects of 4HR on the expressions of NFkB signaling proteins in HUVECs

4HR had different effects on the expressions of nuclear factor kappa-light-chain-enhancer of activated B cells (NFkB) signaling proteins in HUVECs. The expression of NFkB was slightly decreased by 6.2% at 24 h versus nontreated controls, while the expressions of ikappaB kinase (IKK), p38, and p-p38, negative regulators of NFkB function, were increased by 9.3% at 16 h, 15.8% at 16 h, and 12.2% at 8 h, respectively.

**Fig. 2:**
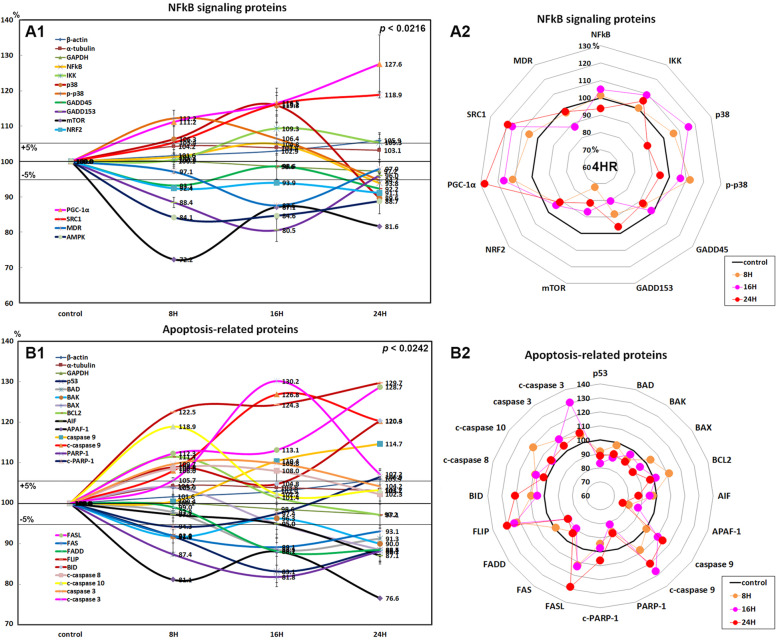
Expressions of NFkB signaling proteins (**A1**, **A2**) and apoptosis-related proteins (**B1**, **B2**) in 4HR-treated HUVECs as determined by IP-HPLC. The line graphs (**A1**, **B1**) show protein expression patterns on the same scale (%) versus culture time (8, 16, or 24 h), whereas the star plots (**A2**, **B2**) show the differential expression levels of proteins in a circular manner after 8, 16, or 24 h of treatment on appropriate scales (%)

4HR diffusely decreased the expressions of downstream NFkB signaling proteins such as growth arrest and DNA damage 45 (GADD45; 7.8% at 24 h), GADD153 (19.5% at 16 h), mammalian target of rapamycin (mTOR; 27.8% at 8 h), nuclear factor (erythroid-derived 2)-like 2 (NRF2; 8.9% at 24 h), multiple drug resistance (MDR; 12.5% at 16 h), and 5′ AMP-activated protein kinase (AMPK; 15.9% at 8 h), but it increased the expressions of peroxisome proliferator-activated receptor gamma coactivator 1-α (PGC-1α; 27.6% at 24 h) and steroid receptor coactivator-1 (SRC1; 18.9% at 24 h; Fig. 2A1, A2).

These results indicate that 4HR significantly suppressed NFkB signaling in HUVECs through upregulation of negative regulators and downregulation of multiple downstream effector proteins.

### Effects of 4HR on the expressions of apoptosis-related proteins in HUVECs

4HR affected the expressions of p53-mediated apoptosis-related proteins, particularly p53 protein, which was decreased by 16.9% after treatment for 16 h as compared with nontreated controls, and decreased the expressions of pro-apoptotic proteins, BCL2-associated death promoter (BAD; 11.5% at 16 h), BCL2 homologous antagonist/killer (BAK; 10% at 24 h), apoptosis regulator BAX (11.5% at 24 h), and apoptotic protease activating factor 1 (APAF-1; 23.4% at 24 h) but increased the expression of B cell lymphoma 2 (BCL2; 11.4% at 8 h) and apoptosis inducing factor (AIF; 13.5% at 8 h). On the other hand, the expressions of apoptosis executor proteins such as caspase 9 and c-caspase 9 were increased by 13.9% at 24 h and by 19% at 16 h, respectively (Fig. 2B1, B2). These results indicate that 4HR activated caspase 9 and c-caspase 9 via AIF signaling in the lack of upregulation of pro-apoptotic factors, including BAD, BAK, BAX, and APAF-1.

HUVECs treated with 4HR showed decreases in the expressions of FAS-mediated apoptosis signaling proteins as compared with nontreated controls, although they showed an increase in the expression of FAS ligand (FASL; 28.7% at 24 h). After treatment with 4HR for 24 h, the expression of death receptors on cell surfaces, that is, FAS, was decreased by 10.9% at 16 h and that of FAS-associated protein with death domain (FADD) was also decreased by 11.9% at 16 h, but FLICE-like inhibitory protein (FLIP) expression was increased by 29.7% at 24 h, whereas HUVECs treated with 4HR showed increases in the expressions of apoptosis executor proteins, c-caspase 8 (by 8.6% at 8 h), c-caspase 10 (18.9% at 8 h), caspase 3 (by 9.8% at 16 h), and c-caspase 3 (by 30.2% at 16 h), and BH3 interacting-domain death agonist (BID; 20.4% at 24 h; Fig. 2)B1, B2). These results indicate 4HR activated caspase 8, caspase 10, and caspase 3 independently from FAS-mediated signaling proteins.

On the other hand, HUVECs treated with 4HR showed decreases in the expressions of poly-[ADP-ribose] polymerase 1 (PARP-1; 18.2% at 16 h) and cleaved PARP-1 (c-PARP-1; 5.7% at 8 h; Fig. 2)B1, B2). These results indicate 4HR rarely produced single-strand DNA breaks, which require repair by PARP-1.

### Effects of 4HR on the expressions of inflammatory proteins in HUVECs

**Fig. 3:**
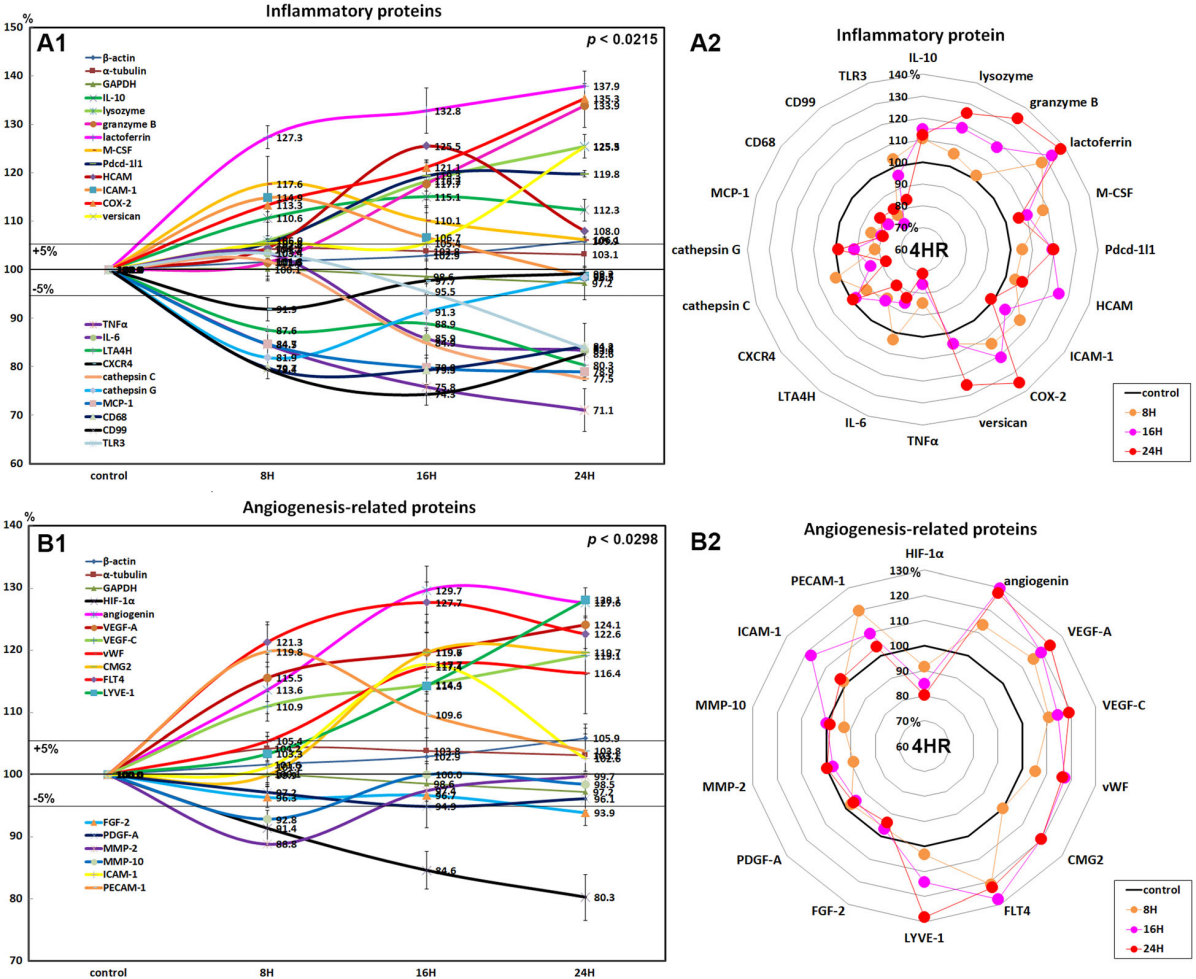
Expressions of inflammatory proteins (**A1**, **A2**) and angiogenesis-related proteins (**B1**, **B2**) in 4HR-treated HUVECs as determined by IP-HPLC. The line graphs (**A1**, **B1**) show protein expression patterns on the same scale (%) versus culture time (8, 16, or 24 h), whereas the star plots (**A2**, **B2**) showed the differential expression levels of proteins in a circular manner after 8, 16, or 24 h of treatment on appropriate scales (%)

4HR influenced the expressions of inflammatory proteins positively or negatively in HUVECs depending on the types of M1/M2 macrophage polarization. The proteins upregulated by 4HR usually belong to M2 macrophage polarization proteins, which were interleukin-10 (IL-10; 15.1% at 16 h), lysozyme (25.5% at 24 h), granzyme B (33.9% at 24 h), lactoferrin (37.9% at 24 h), macrophage colony-stimulating factor (M-CSF; 17.6% at 8 h), programmed cell death protein 1 (Pdcd-1/1, CD279; 19.8% at 24 h), homing cell adhesion molecule (HCAM, CD44; 25.5% at 16 h), intercellular adhesion molecule 1 (ICAM-1, CD54; 14.9% at 8 h), cyclooxygenase 2 (COX-2; 35.3% at 24 h), and versican (25.3% at 24 h) as compared with nontreated controls. The downregulated proteins by 4HR usually belong to M1 macrophage polarization proteins, which were tumor necrosis factor α (TNFα; 28.9% at 24 h), IL-6 (16.6% at 24 h), leukotriene A4 hydrolase (LTA4H; 19.7% at 24 h), C-X-C chemokine receptor type 4 (CXCR4, CD184; 8.1% at 8 h), cathepsin C (22.5% at 24 h), cathepsin G (18.1% at 8 h), monocyte chemotactic protein-1 (MCP-1; 21.1% at 24 h), CD68 (20.7% at 16 h), CD99 (25.7% at 16 h), and toll-like receptor 3 (TLR3; 16.2% at 24 h; Fig. 3A1, A2).

These results indicate that HUVECs treated with 4HR for 24 h showed upregulation of M2 macrophage polarization proteins (IL-10, lysozyme, granzyme B, lactoferrin, M-CSF, Pdcd-1/1, HCAM, ICAM-1, COX-2, and versican) but downregulation of M1 macrophage polarization proteins (TNFα, IL-6, LTA4H, CXCR4, cathepsin C, cathepsin G, MCP-1, CD68, CD99, and TLR3).

### Effects of 4HR on the expressions of angiogenesis-related proteins in HUVECs

HUVECs treated with 4HR showed increases in the expressions of angiogenesis-related proteins for 24 h, as follows: angiogenin (27.6% at 24 h), VEGF-A (24.1% at 24 h), VEGF-C (19.1% at 24 h), von Willebrand factor (vWF; 17.4% at 16 h), capillary morphogenesis protein 2 (CMG2; 19.7% at 24 h), Fms-related tyrosine kinase 4 (FLT-4; 27.7% at 16 h), lymphatic vessel endothelial hyaluronan receptor 1 (LYVE-1; 28.1% at 24 h), ICAM-1 (17.7% at 16 h), and platelet endothelial cell adhesion molecule-1 (PECAM-1, CD31; 19.8% at 8 h), but they showed decreases in the expressions of hypoxia-inducible factor-1α (HIF-1α; 19.7% at 24 h), fibroblast growth factor-2 (FGF-2; 6.1% at 24 h), PDGF-A (5.1% at 16 h), MMP-2 (11.2% at 8 h), and MMP-10 (7.2% at 8 h; Fig. 3B1, B2).

These results indicate that HUVECs treated with 4HR for 24 h showed dramatic upregulation of cellular angiogenetic proteins including angiogenin, VEGF-A, VEGF-C, vWF, CMG2, FLT4, LYVE-1, ICAM-1, and PECAM-1 independently from angiogenesis transcription factor (HIF-1α) and matrix angiogenetic factors (FGF-2, PDGF-A, MMP-2, and MMP-10).

### Global protein expressions in 4HR-induced HUVECs

**Fig. 4:**
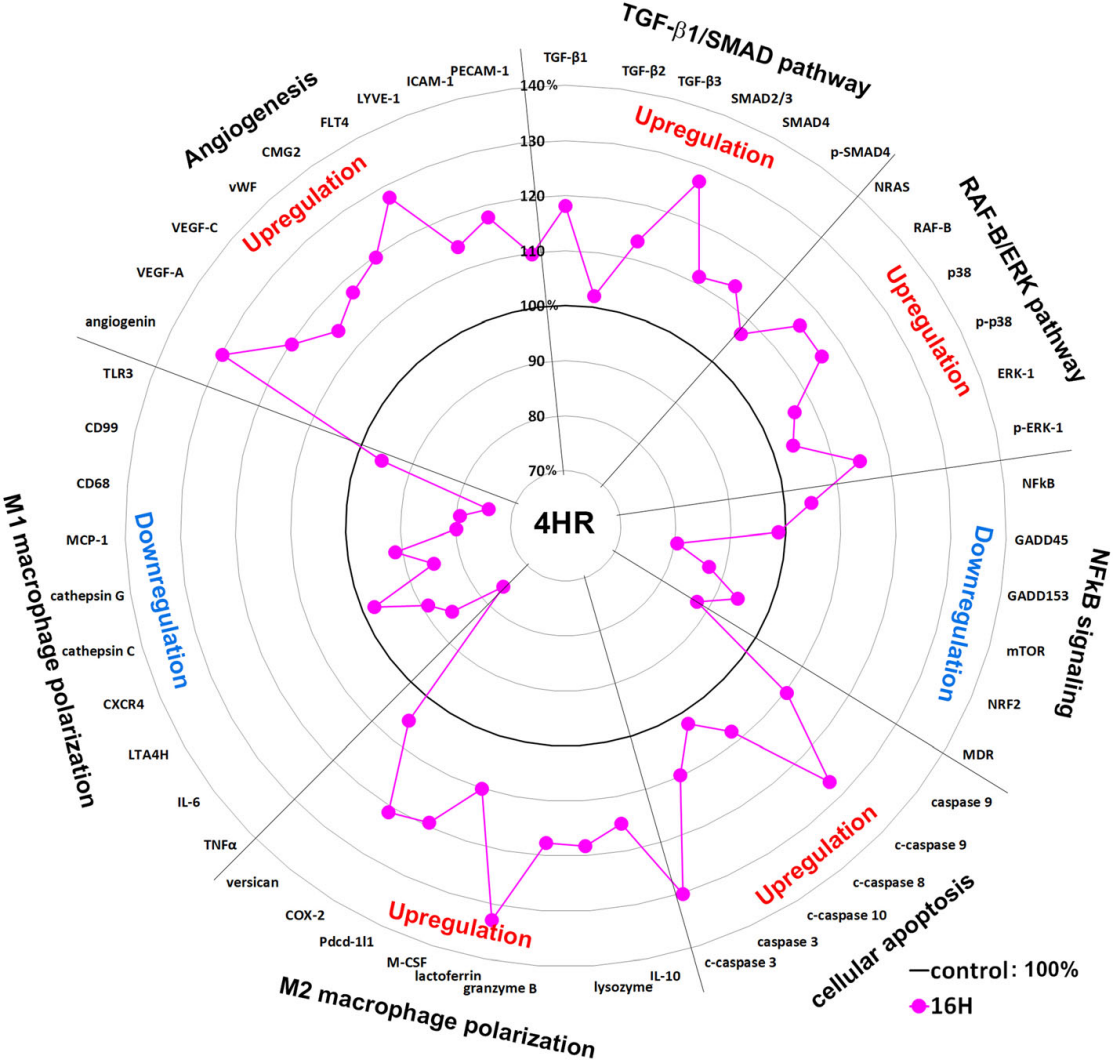
Star plot of global protein expression in HUVECs treated with 4HR for 16 h. The expression levels (%) of representative proteins (*n* = 51) selected from 6 major molecular signaling pathways are plotted in a circular manner. The expressions of growth factor-related proteins (TGF-β/SMAD signaling), RAS signaling proteins (RAF-B/ERK and p38 signaling), cellular apoptosis-related proteins (apoptosis executor proteins; caspase-3, caspase-8, caspase-9, caspase-10), M2 macrophage polarization proteins, and angiogenesis-related proteins were upregulated, while the expressions of NFkB signaling proteins and M1 macrophage polarization proteins were downregulated

Global protein expression changes of representative proteins (*n* = 51) selected from 6 different protein signaling pathways above are illustrated as a star plot in Fig. 4. The growth factor-related proteins (TGF-β1, TGF-β2, TGF-β3, SMAD2/3, SMAD4, and p-SMAD4), RAS signaling proteins (NRAS, RAF-B, p38, p-p38, ERK-1, and pERK-1), cellular apoptosis-related proteins (caspase 9, c-caspase 9, caspase 8, c-caspase 10, caspase 3, and c-caspase 3), M2 macrophage polarization proteins (IL-10, lysozyme, granzyme B, lactoferrin, M-CSF, Pdcd-1/1, COX-2, and versican), and angiogenesis-related proteins (angiogenin, VEGF-A, VEGF-C, vWF, CMG2, FLT4, LYVE-1, ICAM-1, and PECAM-1) were upregulated in HUVECs treated with 4HR for 16 h, while the NFkB signaling proteins (NFkB, GADD45, GADD135, mTOR, NRF2, and MDR) and M1 macrophage polarization proteins (TNFα, IL-6, LTA4H, CXCR4, cathepsin C, cathepsin G, MCP-1, CD68, CD99, and TLR3) were downregulated.

These results indicate that HUVECs treated with 4HR for 16 h may have strong angiogenetic potential with concurrent elevation of TGF-β/SMAD signaling, RAF-B/ERK and p38 signaling, and M2 macrophage polarization and that 4HR-induced activation of caspases and subsequent cellular apoptosis in the reduction of NFkB signaling compensate by stimulating the expressions of TGF-βs in HUVECs.

## Discussion

4HR is a phenolic compound with a hexane chain. Because it is strongly hydrophobic, 4HR can adhere to some proteins and change their molecular conformation into inactive status [24]. In a previous study on the 4HR adhering assay [19], TNFα, lysozyme, PDGF-A, FLT4, pAKT1/2/3, PKC, and GADD45 were significantly adherent to 4HR, but other proteins examined in this study, including growth factor-related proteins (TGF-β1, TGF-β2, TGF-β3, SMADs), RAS signaling proteins (RAF-B/ERK and p38 pathways), NFkB signaling proteins, apoptosis-related proteins (caspase-3, caspase-8, caspase-9, caspase-10), inflammatory proteins (IL-10, M-CSF, COX-2, LTA4H, CXCR4, MCP-1, etc.), and angiogenesis-related proteins (angiogenin, VEGF-A, VEGF-C, CMG2, LYVE-1, etc.), were almost non-adherent or minimal adherent (< 5%) to 4HR-coated beads. These results indicate the expression changes of different proteins observed in this study were due to the active interaction between 4HR and target proteins rather than the direct denaturation of target proteins by 4HR adherence. Therefore, these results suggested that the different molecular signaling by 4-HR in HUVECs be reproducible and devoid of further oxidative stresses or endoplasmic reticulum stresses.

4HR-treated HUVECs showed alternative cellular apoptosis caused by activation of different caspases (cleaved caspase-3, caspase-8, caspase-9, and caspase-10) despite the reduction of p53- and FAS-mediated pro-apoptotic signaling. Although the present study did not elucidate whether 4HR could damage the mitochondrial membrane, it was thought that the relatively innocuous 4HR, which did not elicit any oxidative stress in cells [19], produced abortive mitochondrial biogenesis by upregulation of PGC-1α and AIF but downregulation of AMPK (energy consumption) simultaneously in HUVECs resulting in alternative apoptosis by activation of caspases released from 4HR-involved mitochondria.

This 4HR-induced cellular apoptosis would be slowly progressed with no activation of NFkB signaling and compensate by stimulating TGF-β production in HUVECs. Actually, in the present study, 4HR-treated HUVECs showed dominant expressions of TGF-β1, TGF-β2, and TGF-β3 despite consistent downregulation of FGF-1, FGF-2, FGF-7, growth hormone, growth hormone releasing hormone, PDGF-A, and c-erbB-2 (HER2; some data not shown). The dominant expressions of TGF-β1, TGF-β2, and TGF-β3 were very characteristic in 4HR-treated HUVECs. However, when TGF-β ligands bind to TGF-β receptors (heteromeric complex of type I and type II TGF-β receptors), it is expected that the SMAD2/3/4 pathway is activated and undergoes target gene transcription such as VEGFs and BMPs and that RAF-B/ERK and p38 signaling are activated and cross-talk with TGF-β/SMAD signaling. These TGF-β signaling cascades were found as marked upregulation and activation of RAF-B, SMADs, ERK-1, p38, and VEGFs in the present 4HR-treated HUVECs. In addition, the administration of A83-01, a Smad pathway blocker, showed marked reduction of VEGF expression, which was expected to be increased by 4HR treatment.

In the previous study, we found that 4HR induced potent de novo angiogenesis in both in vitro and in vivo experiments [10]. 4HR treatment increased VEGF expression in RAW264.7 cells, and it is HIF independent. The present study explored the molecular mechanism of 4HR-induced angiogenesis in HUVECs and observed that 4HR-treated HUVECs showed dominant expressions of TGF-βs concurrently with upregulation of SMADs and VEGFs. In addition, it has been confirmed that TGF-β1 stimulates SMAD pathway and increases VEGF-A expression in in vitro culture of HUVECs. Therefore, it is suggested that 4HR-induced angiogenesis in HUVECs is characteristic with serial activation of cellular angiogenetic factors in the TGF-β/SMAD/VEGF pathways independent from the ordinary angiogenesis transcription factor (HIF-1α) and matrix angiogenetic factors (FGF-2, PDGF-A, MMP-2, and MMP-10).

On the other hand, 4HR-treated HUVECs expressed a higher level of M2 macrophage polarization proteins (cytokines) than nontreated controls and a lower level of M1 macrophage polarization proteins. The upregulation of M2 macrophage polarization cytokines might autonomously stimulate HUVECs to undergo cytological changes appropriate for angiogenesis, subsequently followed by HUVEC differentiation via TGF-β/SMAD/VEGF signaling in vitro. Our previous study reported that 4HR induced a strong wound-healing effect with de novo angiogenesis associated with M2 macrophage infiltration in in vivo animal experiments [25, 26]. In this study, however, the HUVEC culture contained no macrophages, so there was no cellular interaction between HUVECs and macrophages, resulting in a diminished angiogenetic effect of M2 macrophage polarization cytokines on HUVECs. Among 4HR-induced angiogenic effects, M2 macrophage polarization proteins will be more greatly amplified in in vivo animal experiments, where macrophages can be infiltrated, than in in vitro cell culture. In addition, 4HR can increase the expression level of M2 markers in RAW264.7 cells directly [10].

**Fig. 5:**
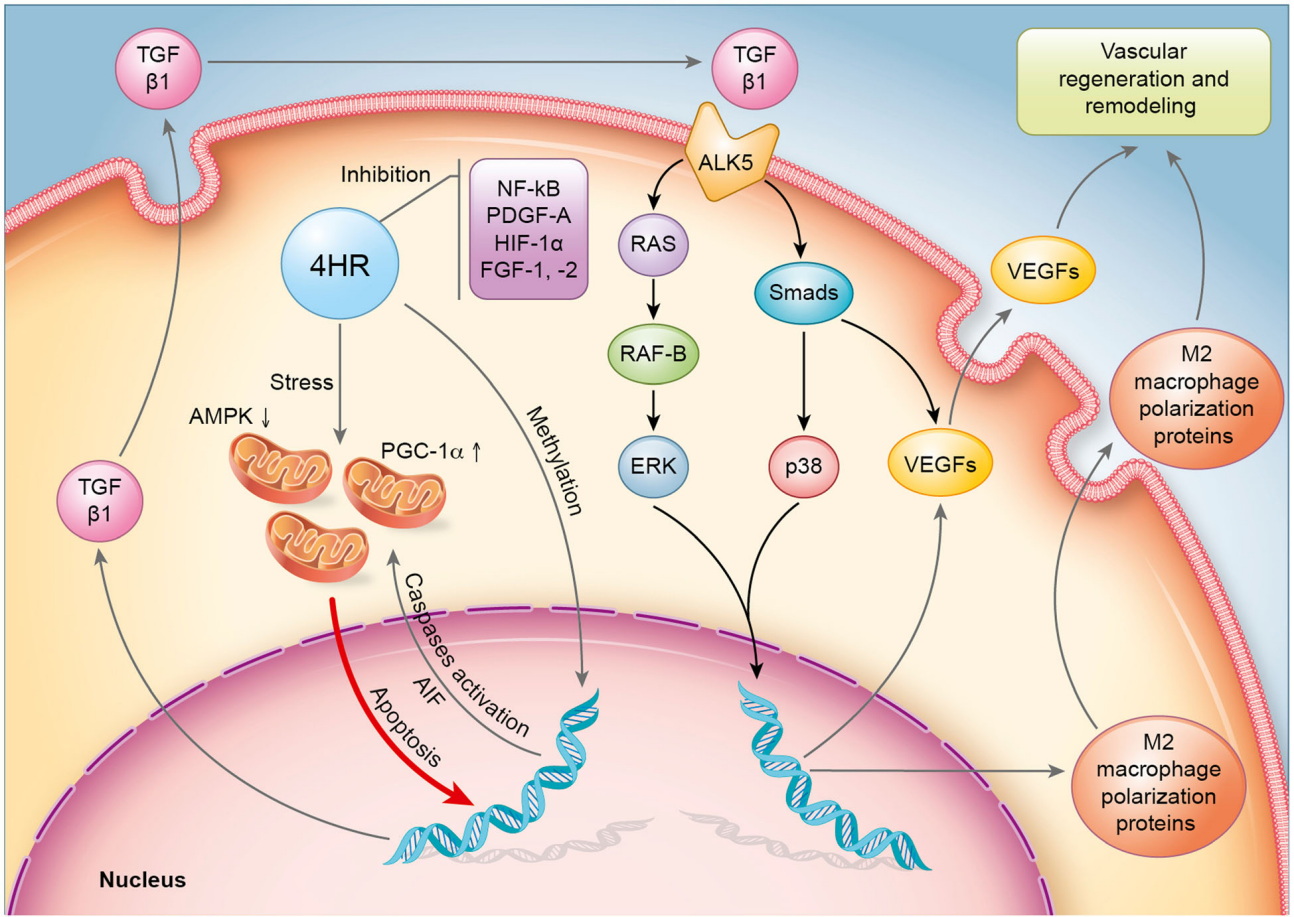
Schematic drawings for the proposed mechanism. Apoptotic stress on the mitochondria is induced by application of 4HR. This stress induces TGF-β1 expression, and secreted TGF-β1 protein will bind to ALK5. Then, the downstream signal is generated by the RAS/Smads pathway. This signal will increase the expression of VEGFs

The global protein expression changes are illustrated in Fig. 4 using 51 representative proteins selected from 6 major molecular signaling pathways. It was found that 4HR-treated HUVECs showed concurrent upregulation of TGF-β/SMAD/VEGF signaling, RAF-B/ERK and p38 signaling, and M2 macrophage polarization and that 4HR-induced activation of caspases and subsequent cellular apoptosis were closely relevant to the overexpression of TGF-βs in HUVECs. If the protein expression patterns obtained from precision IP-HPLC analysis were similar to each other at their level (%) and time after 4HR treatment, the concurrent protein expression changes in the same functional groups may have implications for the signal transduction or cross-talk to achieve the final goals of objective proteins. Therefore, two major angiogenetic pathways induced by 4HR were identified from the above global protein expression data analyzed by IP-HPLC: the caspase activation/TGF-β/SMAD/VEGF pathway and reduced NFkB signaling/upregulation of M2 macrophage polarization proteins/endothelial cell differentiation in HUVECs (Fig. 5).

Angiogenesis is a vital step for uneventful wound healing. The materials inducing M2 macrophage polarization are required for angiogenesis and wound remodeling [27]. 4HR is an agent for M2 macrophage polarization [10]. In this study, 4HR increased the expression level of proteins that are responsible for endothelial cell differentiation (Fig. 5). In future perspective, 4HR incorporating materials may be developed for the maxillofacial regeneration. Actually, 4HR incorporated xenograft has been shown reduced foreign body reaction [28] and accelerated degradation [29]. Bone grafts with 4HR suppress NFkB signaling and increase bone regeneration [30].

## Conclusion

Collectively, 4HR-induced angiogenic factors (VEGFs) were controlled by TGF-β1 overexpression and subsequent activation of SMADs/VEGFs, RAF-B/ERK and p38 signaling, and M2 macrophage polarization. Therefore, it is assumed that 4HR activates TGF-β/SMAD/VEGF signaling and induced vascular regeneration and remodeling for wound healing. In particular, the overexpression of TGF-βs in 4HR-treated HUVECs might be ascribed to the increase of apoptosis via FAS-mediated signaling, and the dominant TGF-β1 expression might induce the protein expressions of M2 macrophage polarization proteins, which subsequently stimulate wound-healing procedures.

## Data Availability

Not applicable.

## Abbreviations

4HR: 4-Hexylresorcinol
VEGF: Vascular endothelial growth factor
MMP: Matrix metalloproteinase
TGF-β1: Transforming growth factor-β1
IP-HPLC: Immunoprecipitation high-performance liquid chromatography
ELISA: Enzyme-linked immunosorbent assay
HUVECs: Human umbilical vein endothelial cells

## References

[1] McConnell VF (1953). Hlasiwetz and Barth - pioneers in the structural aspects of plant products. J Chem Educ.

[2] Wilson CO, Gisvold O, Doerge RF (1966). Textbook of organic medicinal and pharmaceutical chemistry.

[3] He J, Zhu Q, Dong X, Pan H, Chen J, Zheng ZP (2017). Oxyresveratrol and ascorbic acid O/W microemulsion: preparation, characterization, anti-isomerization and potential application as antibrowning agent on fresh-cut lotus root slices. Food Chem.

[4] Darlow HM, Powell EO, Bale WR, Morris EJ (1958). Observations on the bactericidal action of hexyl resorcinol aerosols. J Hyg.

[5] Brkic D (1956). Intestinal parasites: helminths; with special reference to newer antihelminthics. Srp Arh Celok Lek.

[6] Ujiie T (1968). Experimental anticancer studies. XXXIV. Some compounds relating to 4-n-hexyl-6-(2-hydroxyphenyliminomethyl) resorcinol and their anticancer activity. Chem Pharm Bull.

[7] Chevalier M, Sakarovitch C, Precheur I, Lamure J, Pouyssegur-Rougier V (2015). Antiseptic mouthwashes could worsen xerostomia in patients taking polypharmacy. Acta Odontol Scand.

[8] Yen GC, Duh PD, Lin CW (2003). Effects of resveratrol and 4-hexylresorcinol on hydrogen peroxide-induced oxidative DNA damage in human lymphocytes. Free Radic Res.

[9] Guandalini E, Ioppolo A, Mantovani A, Stacchini P, Giovannini C (1998). 4-Hexylresorcinol as inhibitor of shrimp melanosis: efficacy and residues studies; evaluation of possible toxic effect in a human intestinal in vitro model (Caco-2); preliminary safety assessment. Food Addit Contam.

[10] Jo YY, Kim DW, Choi JY, Kim SG (2019). 4-Hexylresorcinol and silk sericin increase the expression of vascular endothelial growth factor via different pathways. Sci Rep.

[11] Liu Y, Zhang H, Yan L, Du W, Zhang M, Chen H, Zhang L, Li G, Li J, Dong Y, Zhu D (2018). MMP-2 and MMP-9 contribute to the angiogenic effect produced by hypoxia/15-HETE in pulmonary endothelial cells. J Mol Cell Cardiol.

[12] Gresele P, Falcinelli E, Momi S (2008). Potentiation and priming of platelet activation: a potential target for antiplatelet therapy. Trends Pharmacol Sci.

[13] Clarke NJ, Tomlinson AJ, Ohyagi Y, Younkin S, Naylor S (1998). Detection and quantitation of cellularly derived amyloid beta peptides by immunoprecipitation-HPLC-MS. FEBS letters.

[14] Luo L, Shen L, Sun F, Ma Z (2013). Immunoprecipitation coupled with HPLC-MS/MS to discover the aromatase ligands from Glycyrrhiza uralensis. Food Chem.

[15] Kim YS, Lee SK (2015). IP-HPLC analysis of human salivary protein complexes. Kor J Oral Maxillofac Pathol.

[16] Kim SM, Eo MY, Cho YJ, Kim YS, Lee SK (2018). Immunoprecipitation high performance liquid chromatographic analysis of healing process in chronic suppurative osteomyelitis of the jaw. J Craniomaxillofac Surg.

[17] Kim SM, Eo MY, Cho YJ, Kim YS, Lee SK (2017). Wound healing protein profiles in the postoperative exudate of bisphosphonate-related osteonecrosis of mandible. Eur Arch Otorhinolaryngol.

[18] Kim SM, Eo MY, Cho YJ, Kim YS, Lee SK (2017). Differential protein expression in the secretory fluids of maxillary sinusitis and maxillary retention cyst. Eur Arch Otorhinolaryngol.

[19] Kim MK, Yoon CS, Kim SG, Park YW, Lee SK (2019). Effects of 4-hexylresorcinol on protein expressions in RAW 264.7 cells as determined by immunoprecipitation high performance liquid chromatography. Sci Rep.

[20] Yoon CS, Kim MK, Kim YS, Lee SK (2018). In vitro protein expression changes in RAW 264.7 cells and HUVECs treated with dialyzed coffee extract by immunoprecipitation high performance liquid chromatography. Sci Rep.

[21] Kim SM, Jeong D, Kim MK, Lee SS, Lee SK (2017). Two different protein expression profiles of oral squamous cell carcinoma analyzed by immunoprecipitation high-performance liquid chromatography. World J Surg Oncol.

[22] Yoon CS, Kim MK, Kim YS, Lee SK (2018). In vivo protein expression changes in mouse livers treated with dialyzed coffee extract as determined by IP-HPLC. Maxillofac Plast Reconstr Surg.

[23] Choi KH, Kim DW, Lee SK, Kim SG, Kim TW (2020). The administration of 4-hexylresorcinol accelerates orthodontic tooth movement and increases the expression level of bone turnover markers in ovariectomized rats. Int J Mol Sci.

[24] Feng S, Song XH, Zeng CM (2012). Inhibition of amyloid fibrillation of lysozyme by phenolic compounds involves quinoprotein formation. FEBS letters.

[25] Ahn J, Kim SG, Kim MK, Kim DW, Lee JH, Seok H, Choi JY (2016). Topical delivery of 4-hexylresorcinol promotes wound healing via tumor necrosis factor-alpha suppression. Burns.

[26] Kang YJ, Noh JE, Lee MJ, Chae WS, Lee SY, Kim SG (2016). The effect of 4-hexylresorcinol on xenograft degradation in a rat calvarial defect model. Maxillofac Plastic Reconstr Surg.

[27] Kim SG (2020). Immunomodulation for maxillofacial reconstructive surgery. Maxillofac Plast Reconstr Surg.

[28] Kweon H, Kim SG, Choi JY (2014). Inhibition of foreign body giant cell formation by 4-hexylresorcinol through suppression of diacylglycerol kinase delta gene expression. Biomaterials.

[29] Jo YY, Kweon H, Kim DW, Kim MK, Kim SG, Kim JY, Chae WS, Hong SP, Park YH, Lee SY, Choi JY (2017). Accelerated biodegradation of silk sutures through matrix metalloproteinase activation by incorporating 4-hexylresorcinol. Sci Rep.

[30] Song JY, Kim SG, Park NR, Choi JY (2018). Porcine bone incorporated with 4-hexylresorcinol increases new bone formation by suppression of the nuclear factor kappa B signaling pathway. J Craniofac Surg.

